# Activity of sorghum aphid and its natural enemies in the context of agroecological and weather conditions

**DOI:** 10.3389/finsc.2025.1503044

**Published:** 2025-02-10

**Authors:** Tomasz E. Koralewski, Michael J. Brewer, Leonel L. Deleon, Norman C. Elliott, Kristopher Giles, Adrianna Szczepaniec, Ashleigh M. Faris

**Affiliations:** ^1^ Department of Entomology, Texas A&M AgriLife Research, Corpus Christi, TX, United States; ^2^ Department of Ecology and Conservation Biology, Texas A&M University, College Station, TX, United States; ^3^ Peanut and Small Grains Research Unit, Agricultural Research Service, United States Department of Agriculture, Stillwater, OK, United States; ^4^ Department of Entomology and Plant Pathology, Oklahoma State University, Stillwater, OK, United States; ^5^ Department of Agricultural Biology, Colorado State University, Fort Collins, CO, United States

**Keywords:** biological control, ecosystem services, invasive species management, *Melanaphis sorghi*, natural enemies, sorghum, sorghum aphid, sugarcane aphid

## Abstract

Agroecological-oriented areawide pest management leverages the innate ability of agroecosystem to suppress pests, and thus to utilize ecosystem services, a key component of sustainable agriculture. A growing body of knowledge on interactions between pests and their natural enemies allows us to recognize the complexity of these interactions that often depend on environmental circumstances. Sorghum aphid, *Melanaphis sorghi* (Theobald) (Hemiptera: Aphididae), is a recent but established pest of sorghum in the Great Plains of North America. Both predators and parasitoids prey on sorghum aphid but their activity and impact change throughout the area and throughout the year. Both landscape and weather factors have been shown to affect the abundance and numerical responses of these insects, consistent with observations in other aphid species. In this study we used data on counts of sorghum aphids, lady beetles (Coleoptera: Coccinellidae), and parasitoid wasps *Aphelinus nigritus* Howard (Hymenoptera: Aphelinidae) and *Lysiphlebus testaceipes* (Cresson) (Hymenoptera: Braconidae) collected in Kansas, Oklahoma and Texas states of the United States. We analyzed insect dynamics in the context of landscape and weather factors. We built multiple regression models using data from the years 2017–2019 for metrics such as maximum number of insects per leaf, response time of natural enemies to pest presence, and speed of increase in insect abundance. Our results indicate that various aspects of landscape composition, landscape configuration, and weather affect various insect groups and various aspects of insect dynamics in the field. Moreover, characteristics of specific landscape categories seemed to be more informative than overall measure of landscape diversity. Our study provides insights on interactions along both spatial and temporal scales, with the latter considered understudied.

## Introduction

The foundation of agroecological-oriented areawide pest management (AWPM) applied to crop protection is the recognition that pest suppression is innate to the agroecosystem. This innate ability to suppress pests is supported by specific pest management tactics that are regionally applied (1). An operational distinction between classical pest management tactics and AWPM is that the classical tactics tend to focus on individual fields (2), whereas AWPM highlights spatial variability of agricultural and environmental conditions that influence pests (1). The underlying premise of AWPM is that many economic pests of agriculture are more effectively managed using pest management tactics applied in a coordinated strategy over a large area than by using a more traditional approach where tactics are applied to individual fields without considering pest management synergies areawide (3).

Natural enemies are an important pillar of the agroecosystem response (4) but their effectiveness in controlling pests may be impacted by agricultural processes and weather conditions. For example, extensive monocultures in agroecological landscape could be expected to negatively impact populations of natural enemies, although the relationship is not straightforward due to complex processes and interactions in the field and indirect effects of other factors, such as timing (5). Habitat manipulation in direct neighborhoods of crop fields is thought to have the potential to counteract these negative processes but in-depth knowledge of the system as a whole and of its components individually is often insufficient (6). Populations of natural enemies and the biological control services they provide are often affected by grain size of the landscape, with fine grained landscapes generally providing greater biological control of crop pests than coarse grained landscapes (7). In addition to effects of grain size, connectivity of the landscape for natural enemies can be increased by the presence of corridors of acceptable habitat and by field edges both of which can serve as habitat and conduits for dispersal of natural enemies across the landscape (7, 8). A robust general rule may not be feasible as various aspects of landscape structure may have different effects on various components of natural pest control (9), and these effects can vary both spatially and temporally (7, 10).

Invasive aphid, *Melanaphis sorghi* (Theobald) [sorghum aphid; previously published as sugarcane aphid, *Melanaphis sacchari* (Zehntner) (Hemiptera: Aphididae) (11)] was recognized as a major pest of sorghum [*Sorghum bicolor* (L.) Moench (Poales: Poaceae)] in the Great Plains of North America in 2013 (12). It has since become a persistent pest of sorghum in nearly all sorghum producing regions of the United States, with invasions recurring annually. The aphid may cause substantial economic loss through negative impact on sorghum plants and through negative impact on harvesting efficiency due to sticky honeydew excretion buildup on leaves (12, 13) but the impact can vary regionally with climate and types of sorghum grown (14). Multiple aspects of the aphid have been studied, including its biology (15, 16), distribution (17), dispersal (18), economic impact (13, 19), and forecasting (20, 21). Areawide pest management has been proposed as a proper strategy to manage the pest (14). A promising, intensely explored, research avenue in the management of sorghum aphid focuses on the natural enemies of sorghum aphid (10, 22–25), consistent with agroecological-oriented AWPM (1).

In the context of the most recent invasions, studies on natural enemies of sorghum aphid have focused on two important functional groups, predators and parasitoids. These species were studied in the context of environmental factors, such as climate, weather and landscape. Faris et al. (23) identified 19 predatory and parasitoid species preying on sorghum aphid and showed that these natural enemies may reside in Johnson grass [*Sorghum halepense* (L.) Pers.] and in riparian areas during off-season. Additionally, Faris et al. (24) showed that suppression of the aphid by natural enemies is effective on both resistant and susceptible sorghum hybrids. Elkins et al. (10) found that both landscape composition and configuration were associated with the level of sorghum aphid and natural enemy abundance, and higher landscape complexity had negative impact on the numerical response of parasitoids, but not on the numerical response of predators. Despite annual variability of their results, they showed that various aspects of aphid and natural enemy dynamics may need to be considered more holistically. On a larger spatial scale, on which agro-landscape and weather metrics show greater differentiation, the relationships between these metrics and natural enemy dispersal, density, and population growth may vary by region, with landscape composition likely impacting some natural enemy dynamics (11). These studies are important in-depth contributions to the growing body of knowledge on natural pest control, and more broadly, on leveraging ecosystem services in the agroecological context (9, 26–28). Nevertheless, longitudinal data is needed to better understand the dynamics of top-down control of the population of pests (29).

In this study we focus on sorghum aphid and its natural enemies, predatory lady beetles (Coleoptera: Coccinellidae) and parasitoid wasps *Aphelinus nigritus* Howard (Hymenoptera: Aphelinidae) and *Lysiphlebus testaceipes* (Cresson) (Hymenoptera: Braconidae) due to their widespread presence and importance to sorghum aphid population dynamics (25). Building on a major data collection effort over 5 years and three US states, we use statistical methods to investigate several spatiotemporal aspects of insect population dynamics. We relate those aspects to environmental characteristics of sorghum fields, the primary habitat of sorghum aphid, the surrounding landscapes, and local weather conditions. The findings will define spatiotemporal attributes with significant implications to sorghum aphid AWPM on the Great Plains.

## Materials and methods

### Study area and insect sampling

**Figure 1:**
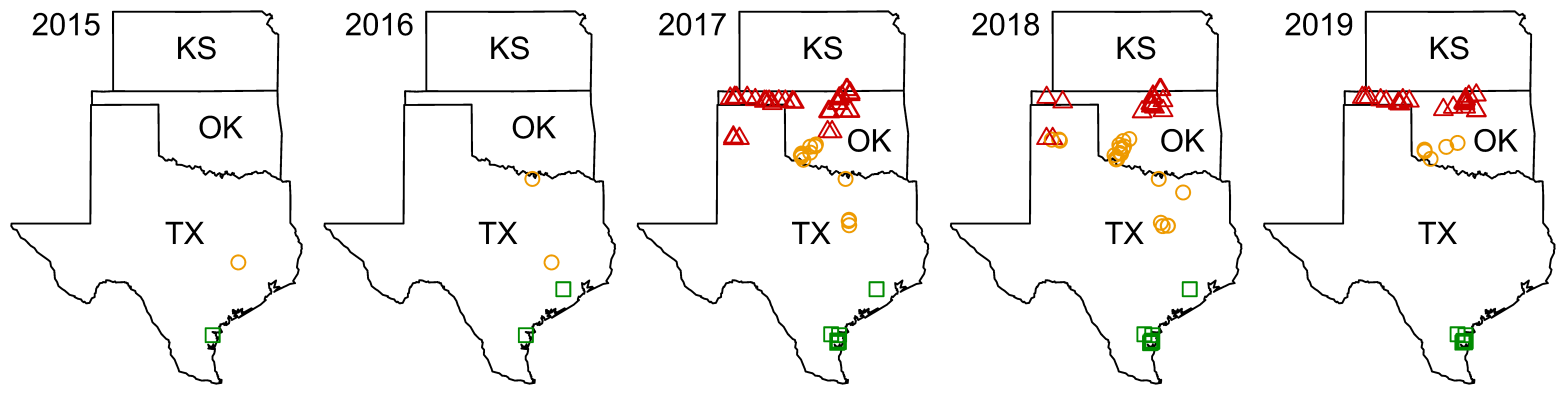
Locations of sampling sites during the years 2015–2019. Markers indicate the locations of sampling sites within the two areas of North American Great Plains and in its southern extension following Brewer et al. (11): red triangles – North Great Plains (N GP), orange circles – South Great Plains (S GP), and green squares – South (S).

Data on counts of sorghum aphid and its natural enemies were collected from sites in Kansas (years 2017–2018), Oklahoma (years 2017–2019), and Texas (years 2015–2019) (**Figure 1**, **Supplementary Table S1**). The five-year study spatially overlapped with and temporally followed sorghum aphid’s rapid geographic expansion from 2013 to 2015 (12). Insect sampling included six family-level taxa with species that were confirmed natural enemies of sorghum aphid (30). The focus of this study was on the most abundant members of the Aphelinidae, Braconidae, and Coccinellidae (11). Considering variability in agroecological and climate conditions, three regions were previously delineated in the study area – two overlapping a large part of the North American Great Plains, termed North Great Plains (N GP) and South Great Plains (S GP), and one overlapping its southern extension, termed South (S) (11). Cereal grain production in the area, including sorghum, is intense but variable. Climate in the area varies from subtropical temperate climate of the Rio Grande Valley of southern Texas, through a warm temperate climate of central Texas and central Oklahoma, to a temperate climate of Texas and Oklahoma Panhandles and northern Oklahoma and southern Kansas.

Sorghum fields sampled were randomly selected from a list of fields with probable sorghum aphid infestations within these three regions provided by local collaborators. Data were taken from multiple fields per county and region to capture both within- and between-region variability of sorghum aphid and natural enemy populations. Sampling began when sorghum aphid was first detected. In some cases, average densities exceeded the threshold of 40 aphids per leaf that were indicators of probable sorghum injury and risk to grain production (19). Individual fields were sampled between 1 and 14 times (median 5), with a typical interval of 7 days (median 7). Dates of sampling and approximate GPS coordinates of sampling sites were recorded. For each sampling event, sorghum aphids and natural enemies from each taxon were counted on two leaves of each plant: the first green leaf toward the base of the plant and the uppermost unfurled leaf below the flag leaf. Plants were selected randomly. The length of inspection time per sampling event was variable, with a minimum of ca. 20 min and with additional time spent when aphid and natural enemies were common (11). Between 48 and 1920 leaves were examined on a given sampling event per field (median 108).

Both alate and apterous sorghum aphids were counted and their counts were combined for further analysis. Counts of lady beetle larvae and adults were recorded and the counts were also combined for further analysis. The parasitized aphids (mummies) were periodically reared to adults and identified as *A. nigritus* (blue-black mummies) based on comparison to voucher specimens (30) or as *L. testaceipes* (light-brown mummies) based on taxonomic keys (31). The counts of *A. nigritus* and *L. testaceipes* mummies were combined for further analysis. Throughout the manuscript we refer to these combined specific groups simply as sorghum aphids, lady beetles, and mummies, respectively. Per-leaf insect counts were used in the downstream analysis. Details on sampling procedures and specimen identification are available (11, 25, 30).

### Insect metrics

The spatiotemporal data on counts of sorghum aphids and their natural enemies allowed us to describe various aspects of insect population dynamics in the field. We defined ten metrics to reflect these dynamics as follows: (1) nSAmax – maximum number of sorghum aphids (both alate and apterae) per leaf, (2) nLBmax – maximum number of lady beetles (both juvenile and adult) per leaf, (3) nMMmax – maximum number of mummies (combined counts of putative *A. nigritus* and putative *L. testaceipes*) per leaf, (4) rLBMMmax – ratio of nLBmax and nMMmax, (5) dtSAmax – time (number of days) during which aphid count per leaf increases from zero to maximum (time to maximum aphid count per leaf), (6) dtRespLB – presumed lady beetle response time to sorghum aphid presence, calculated as the number of days from the first day when sorghum aphids were observed after previously not being observed to the first day when lady beetles were observed after previously not being observed, (7) dtRespMM – presumed parasitoid response time to sorghum aphid presence, calculated as the number of days from the first day when sorghum aphids were observed after previously not being observed to the first day when putative *A. nigritus* or *L. testaceipes* mummies were observed after previously not being observed, (8) vSA – increase of the number of sorghum aphids per leaf per day, from the last day when sorghum aphids were not observed to the day when their number reached maximum (speed of increase in sorghum aphid abundance), (9) vLB – increase of the number of lady beetles per leaf per day, from the last day when lady beetles were not observed to the day when their number reached maximum (speed of increase in lady beetle abundance), (10) vMM – increase of the number of putative *A. nigritus* and *L. testaceipes* mummies per leaf per day, from the last day when the mummies were not observed to the day when their number reached maximum (speed of increase in parasitoid abundance). Each value of each metric was estimated based on one time series of sampling events at a given field within a given year. One such value was considered one data point for the purpose of our analysis. For nSAmax, nLBmax, nMMmax, dtRespLB, and dtRespMM, values greater than 0 were included in downstream analysis. Compared to the years 2015–2016, data collected during 2017–2019 were more abundant and more representative of the breadth of the weather and agroecological conditions in the region (**Figure 1**, **Supplementary Table S2**), and therefore in our analysis we focused on the data from 2017–2019.

### Landscape data

Land cover data for Kansas, Oklahoma, and Texas were extracted from the Cropland Data Layer (CDL) provided by the United States Department of Agriculture (USDA) National Agricultural Statistics Service (NASS) (32). Using geographic information system (GIS) software ArcMap v. 10.8.1 (33), circular areas (buffers) were delineated around each site in each year. We used the radius of 5 km to account for both the preference of smaller area of activity of parasitoids and a larger area of activity of predators (26, 34, 35). The original land cover classes defined in the Cropland Data Layer, and present within the buffers, were reclassified into seventeen composite categories, including one background category (**Supplementary Table S3**). Reclassification accounted for agroecological features significant for sorghum aphid and for its natural enemies, and for the management of sorghum aphid. This step allowed us to reduce the number of landscape metrics considered in our analysis, and thus to focus on meaningful functional relationships across a vast range of agroecological conditions.

### Landscape metrics

To characterize the specificity of the landscape in each buffer, we considered several metrics that describe diverse aspects of composition and configuration of landscape. The metrics were computed in Fragstats v. 4.2 (36) for the reclassified land cover data (**Supplementary Table S3**), and we follow the terminology used in Fragstats. The considered composition metrics included percentage of landscape (PLAND), Simpson’s diversity index (SIDI), and Simpson’s evenness index (SIEI). The considered configuration metrics included patch density (PD), edge density (ED), median shape index (SHAPE_MD), clumpiness index (CLUMPY), and median proximity index (PROX_MD). SIDI and SIEI are landscape-level metrics, whereas all the remaining metrics were computed at the class level. We computed PLAND for each of the 16 landscape categories, and PD, ED, SHAPE_MD, CLUMPY and PROX_MD for sorghum. SHAPE_MD was dropped from further analysis due to the lack of variability in parameter estimates.

Briefly, PLAND describes the percentage of landscape class area within the buffer and is calculated as a ratio of the sum of the areas of all patches of a given class and the total area of the buffer. PD is a configuration/aggregation metric that equals the number of patches of a given class in the buffer per 100 ha. ED is a configuration/edge metric that describes the length of edges of all patches of a given class within the buffer per hectare. SHAPE_MD is a configuration/shape metric that measures shape complexity. CLUMPY is a configuration/aggregation metric that measures class-specific aggregation. PROX_MD is a configuration/aggregation metric that describes the spatial context of a patch relative to its neighbors of the same class with respect to their size and proximity. SIDI measures landscape diversity and represents the probability that any two pixels selected from the buffer at random would be of different categories. SIEI measures how evenly the landscape area is distributed among all classes.

To focus on the most impactful landscape categories in downstream analysis, we jointly considered their ecological relevance to the studied system and the percentage of the area they cover in buffers PLAND (unit: %; 0 < PLAND ≤ 100). We selected five landscape categories: (1) sorghum (the focal crop), (2) wheat, (3) grassland, pasture, and herbaceous, (4) cotton, and (5) woodland. Various crops and grasslands can serve as potential alternative hosts for sorghum aphid and as primary hosts for other aphid species, and thus may support populations of natural enemies (15, 23), whereas the presence of woodland was shown to have an association with the abundance of natural enemies (10, 26). These were also the five most abundant landscape categories in buffers (highest PLAND values) during the years 2017–2019 (**Table 1**). We refer to these respective PLAND metrics as: PLANDs for sorghum, PLANDwh for wheat, PLANDg for grassland, pasture, and herbaceous, PLANDc for cotton, and PLANDwo for woodland. Moreover, we evaluated all remaining landscape metrics for sorghum using Spearman correlation, as implemented in the function cor from the R package stats v. 4.3.2 (37), R v. 4.3.2 (37). Metrics describing sorghum were highly correlated in pairwise comparisons (**Supplementary Table S4**), and therefore, in addition to PLANDs, we retained PD [unit: (100 ha)^–1^; PD > 0, where the maximum value is constrained by spatial resolution and indicates that every other pixel is of the focal class] as a measure of landscape configuration. The landscape-level metrics SIDI and SIEI were also highly correlated, and we retained SIDI (unitless; 0 ≤ SIDI < 1; the value of 0 indicates that the buffer contains only one patch/class, and the value approaches 1 when the number of distinct classes increases, and the area of the buffer is more equally distributed among the classes) for further analysis.

**Table 1:** Land cover categories and median PLAND value [%] for all buffers during the years 2017–2019.

Category	2017	2018	2019
Asteraceae	0.0	0.0	0.0
Brassicaceae	0.0	0.0	0.0
Corn	0.1	0.6	2.1
* Cotton	1.6	4.9	2.7
Fallow	3.0	0.6	1.4
Fruit trees	0.0	0.0	0.0
* Grassland, pasture, and herbaceous	27.4	23.3	13.6
Other crop grasses	0.0	0.0	0.1
Other herbaceous, vegetables, fruits, and field crops	0.0	0.0	0.0
Other Leguminosae	0.1	0.2	0.2
Solanaceae	0.0	0.0	0.0
* Sorghum	3.5	3.9	4.8
Soybean	0.0	0.0	0.0
Wetland	0.1	0.3	0.5
* Wheat	21.2	26.3	19.6
* Woodland	3.1	4.4	6.9

* Categories selected for downstream analysis.

### Weather data and metrics

To evaluate the impact of the weather conditions on the local insect population dynamics, we downloaded daily maximum temperature (TMAX; °C) and precipitation (PPT; mm) records at 4 km resolution for the years 2017–2019 from the PRISM database (38). Daily weather records were retrieved from the PRISM datasets for each site, for the period during which the field data were collected at the site, using the function extract from the R package raster v. 3.6.26 (39). We used four nearest raster cells to interpolate the value at the site location (the parameter method set to “bilinear”). To account for the effects of extreme temperature at each site, we found the maximum value of maximum temperature (maxTMAX; °C) and calculated the standard deviation of maximum temperature (sdTMAX; °C). To account for the effects of precipitation at each site, we calculated mean precipitation (meanPPT; mm) and the coefficient of variation of precipitation (cvPPT).

### Statistical analysis

**Figure 2:**
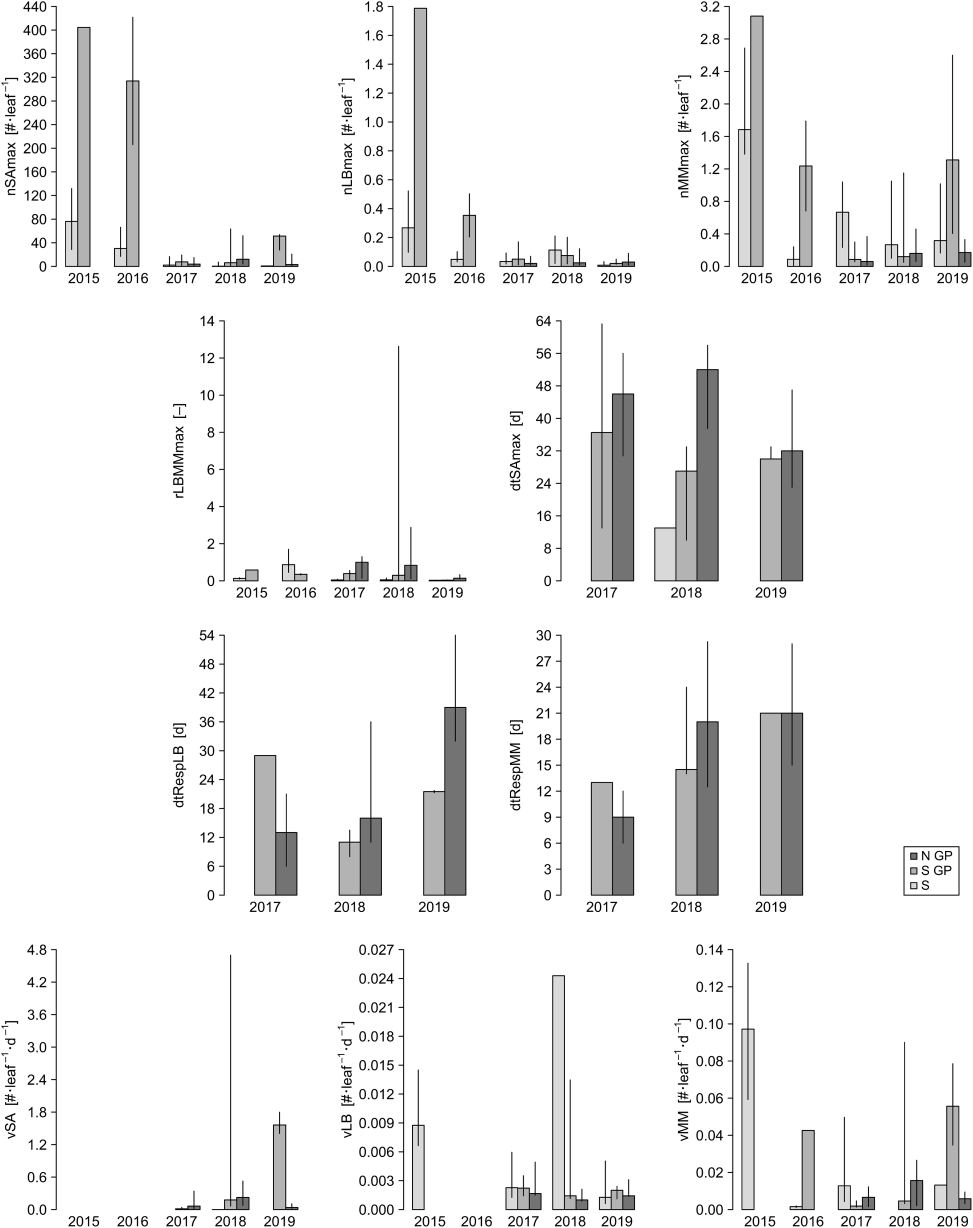
Spatiotemporal distribution of the values of insect population dynamics metrics for the years 2015–2019 for North Great Plains (N GP), South Great Plains (S GP), and South (S). Bars represent medians for the buffers within a given year-region, and vertical lines represent the associated interquartile ranges. Insect population dynamics metrics: nSAmax, maximum number of sorghum aphids per leaf; nLBmax, maximum number of lady beetles per leaf; nMMmax, maximum number of mummies per leaf; rLBMMmax, ratio of nLBmax and nMMmax; dtSAmax, time to maximum aphid count per leaf; dtRespLB, presumed lady beetle response time to sorghum aphid presence; dtRespMM, presumed parasitoid response time to sorghum aphid presence; vSA, speed of increase in sorghum aphid abundance; vLB, speed of increase in lady beetle abundance; vMM, speed of increase in parasitoid abundance.

We evaluated correlation between insect population dynamics metrics using Spearman correlation, as implemented in the function cor from the R package stats v. 4.3.2 (37). We used multiple regression analysis to describe the relationships between each of the ten insect metrics (dependent variables) and the landscape and weather metrics (independent variables) during the years 2017–2019 (**Supplementary Table S5**), with the assumption that the effects of independent variables are additive. Annual variability was high for some of the insect metrics (**Figure 2**). We used ANOVA to determine if the annual differences for each of the ten metrics were statistically significant. The assumptions of ANOVA include random sampling, independence of errors, homogeneity of variance, normality, and additivity of the main effects. In the case of nSAmax, nLBmax, and nMMmax, the anticipated set of independent variables included cvPPT (see below), and the value of cvPPT could not be calculated for some records. We removed these records from the dataset prior to running ANOVA. We ran one-way ANOVA on ranks using the function aov from the R package stats v. 4.3.2 (37). ANOVA results were statistically significant for three metrics, rLBMMmax, dtRespLB, and dtRespMM (**Table 2**), and therefore were followed with a *post-hoc* analysis using Tukey Honest Significant Difference (HSD) test as implemented in the function TukeyHSD from the R package stats v. 4.3.2 (37) that implements an unbalanced design correction. Assumptions of the Tukey HSD test are aligned with those of ANOVA. Based on the results of the Tukey HSD test (**Table 3**), we combined the data from 2017 and 2018 for rLBMMax and dtRespLB, and the data from 2018 and 2019 for dtRespMM. The combined (larger) subsets were used for multiple regression analysis. We combined the data from the years 2017–2019 for the remaining seven metrics: nSAmax, nLBmax, nMMmax, dtSAmax, vSA, vLB, and vMM.

**Table 2:** One-way ANOVA on ranks. Number of data points (n) for each year (2017–2019), combined number of data points for three years [n (all)], and the ANOVA p-value (p).

Metric	n (2017)	n (2018)	n (2019)	n (all)	p
nSAmax	55	47	39	141	0.610
nLBmax	46	34	27	107	0.216
nMMmax	49	34	31	114	0.205
rLBMMmax	43	26	27	96	0.018
dtSAmax	24	28	18	70	0.392
dtRespLB	10	8	7	25	0.016
dtRespMM	8	14	8	30	< 0.001
vSA	24	28	18	70	0.094
vLB	29	20	18	67	0.764
vMM	35	19	18	72	0.105

The nSAmax, nLBmax, and nMMmax records for which cvPPT could not be calculated were removed prior to the analysis.

**Table 3:** Tukey Honest Significant Difference (HSD) test results (p-values) for the pairs of data sets from the years 2017–2019.

Metric	2017–2018	2017–2019	2018–2019
rLBMMmax	0.632	0.079	0.018
dtRespLB	0.940	0.017	0.046
dtRespMM	0.003	< 0.001	0.561

**Figure 3:**
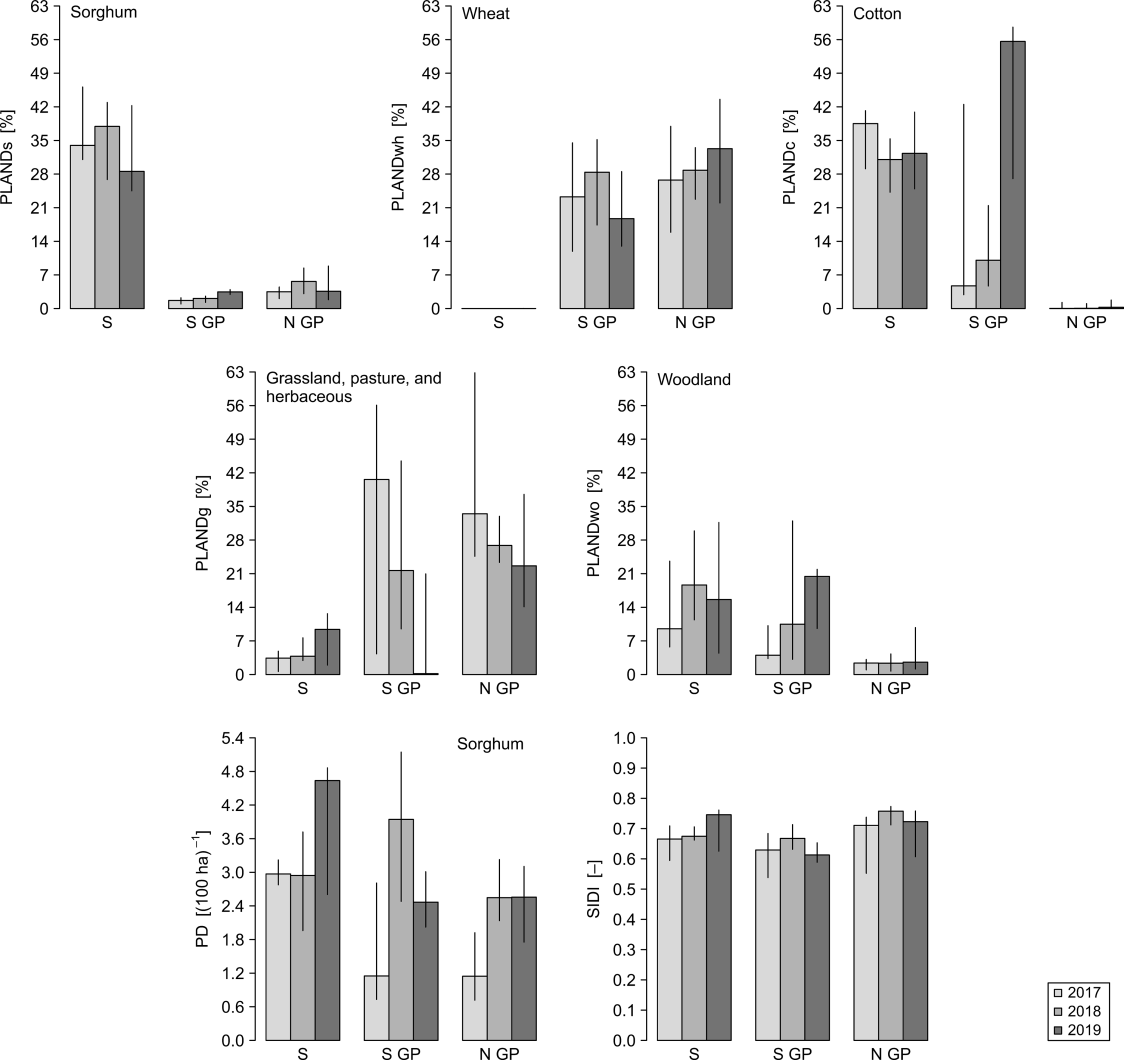
Spatiotemporal distribution of the values of landscape dynamics metrics for the years 2017–2019 for North Great Plains (N GP), South Great Plains (S GP), and South (S). Bars represent medians for the buffers within a given year-region, and vertical lines represent the associated interquartile ranges. Landscape metrics: PLANDs, percentage of landscape (sorghum); PLANDwh, percentage of landscape (wheat); PLANDc, percentage of landscape (cotton); PLANDg, percentage of landscape (grassland, pasture, and herbaceous); PLANDwo, percentage of landscape (woodland); PD, patch density (sorghum); SIDI, Simpson’s diversity index.

**Figure 4:**
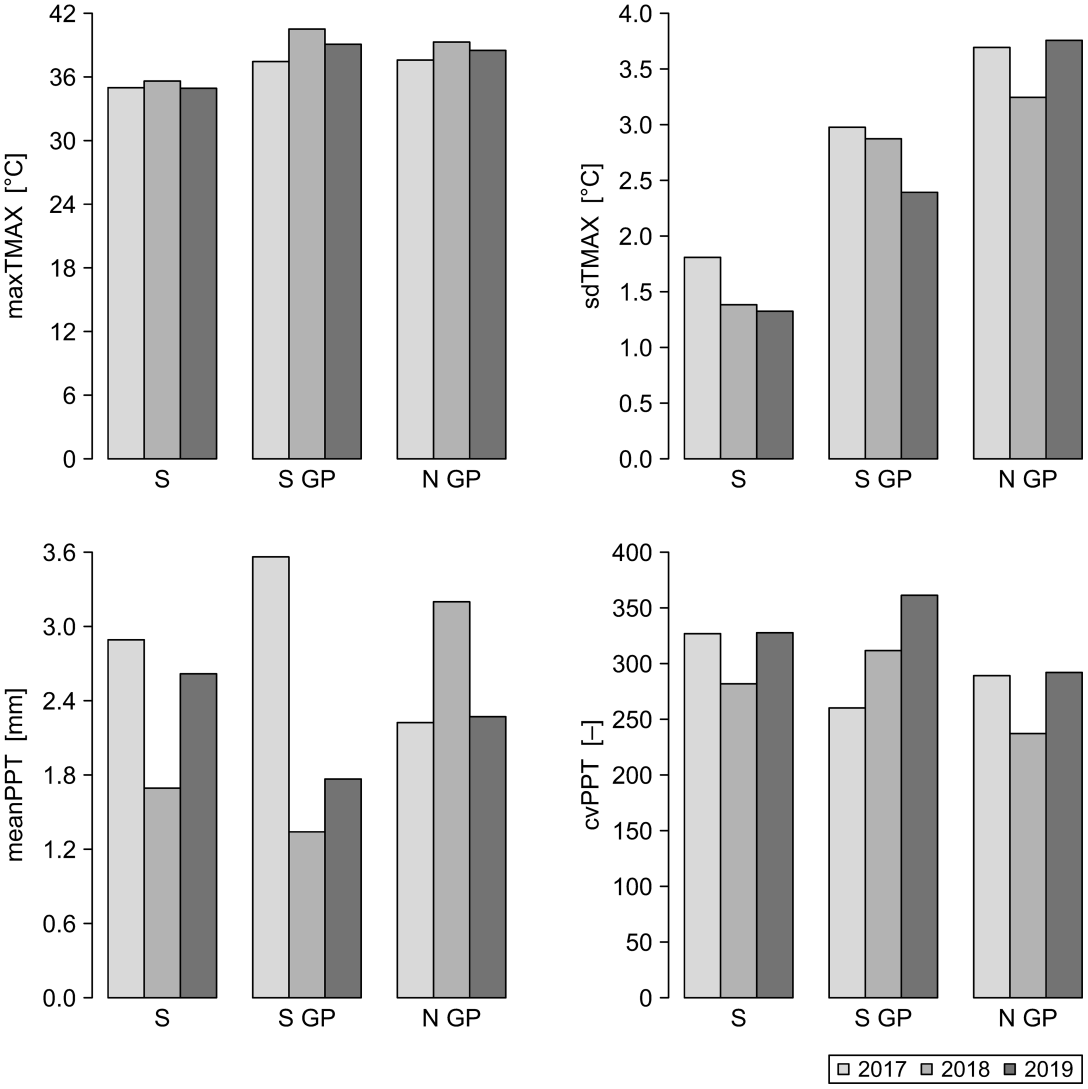
Spatiotemporal distribution of the mean values of weather metrics for the years 2017–2019 for North Great Plains (N GP), South Great Plains (S GP), and South (S). Weather metrics: maxTMAX, maximum value of maximum temperature; sdTMAX, standard deviation of maximum temperature; meanPPT, mean precipitation; cvPPT, coefficient of variation of precipitation.

The independent variables reflected a gradual change in agroecological and weather conditions for the range of locations from coastal to inland (**Figures 3**, **4**). Due to the different number of data points available for each dependent variable, the numbers of independent variables considered for each model varied (**Table 4**). We built multiple regression models for the ten dependent variables using the function lm from the R package stats v. 4.3.2 (37).

**Table 4:** Independent variables (landscape metrics and weather metrics) and dependent variables (insect population dynamics metrics) used in the multiple regression analysis.

Dependent variable	n	PLANDs	PLANDwh	PLANDc	PLANDg	PLANDwo	PD	SIDI	maxTMAX	sdTMAX	meanPPT	cvPPT
ln(nSAmax)	141	o	o	x	v	x	o	x	x	x	o	x
ln(nLBmax)	107	x	x	o	v	x	o	o	o	x	x	x
ln(nMMmax)	114	o	x	o	v	x	x	x	x	x	o	o
ln(rLBMMmax)	69	o	x	x			o	x	x		x	
dtSAmax	70	x	o	x			x	x	x		o	
dtRespLB	18	x						o				
dtRespMM	22	x						x				
ln(vSA)	70	o	x	x			o	x	x		o	
ln(vLB)	67	x	o	o			x	x	x		o	
ln(vMM)	72	x	x	o			o	x	o		x	

Number of data points (n) available for each multiple regression model corresponds to the combined data for the years 2017–2019, except for rLBMMmax (years 2017–2018), dtRespLB (years 2017–2018), and dtRespMM (years 2018–2019). The status of a given candidate independent variable in each multiple regression model is indicated by letters: o – variable included in the optimal (reduced) model with the lowest AICc score, x – variable considered but not included in the optimal model, v – variable removed due to high VIF score (PLANDg only).

After fitting a multiple regression model for each dependent variable, we reviewed the four diagnostic plots to examine assumptions of multiple regression: (1) Residuals vs Fitted plot for linearity of residuals, (2) Normal Q-Q plot for normal distribution of residuals, (3) Scale-Location plot for homoscedasticity, and (4) Residuals vs Leverage plot for influential data points. Analysis of the plots indicated nonlinearity and heteroscedasticity in case of seven dependent variables (nSAmax, nLBmax, nMMmax, rLBMMmax, vSA, vLB, and vMM). We transformed these dependent variables using natural logarithmic transformation (**Table 4**). To evaluate the degree of collinearity among independent variables, we examined the variance inflation factor (VIF) for each full model. We calculated the VIF using the function vif from the R package car v.3.1.2 (40). The VIF reflects the amount of variability of an independent variable explained by other independent variables in the model that can be attributed to correlation among these variables. VIF values that exceed a threshold in the range from 5 to 10 are commonly considered large (41). In the case of nSAmax, nLBmax, and nMMmax, the VIF score for PLANDg was 13.8, 12.0, and 13.0, respectively, and thus PLANDg was not considered further. The recalculated VIF score was less than 3 for each remaining independent variable in each of the ten full models. We then identified optimal (reduced) models for the dependent variables (**Table 5**). We used the function dredge from the R package MuMIn v 1.47.5 (42) to select optimal models based on the Akaike information criterion with a correction for small sample sizes (AICc). We identified the model with the lowest AICc score as the optimal model. None of the candidate models for dtRespLB and for dtRespMM were statistically significant (p ≥ 0.05). For the remaining eight dependent variables, the R^2^ for the optimal models based on AICc score ranged from 0.104 to 0.318, and the adjusted R^2^ ranged from 0.069 to 0.297 (**Table 5**).

**Table 5:** Optimal multiple linear regression models according to the AICc criterion.

Dependent variable	Independent variable	Coefficient estimate	p (coefficient)	R^2^	Adj R^2^	p (model)
ln(nSAmax)	PLANDs PLANDwh PD meanPPT Intercept	–0.0268 0.0376 0.2220 0.4283 –0.7778	0.1408 0.0179 0.0373 0.0005 0.2743	0.151	0.126	0.0002
ln(nLBmax)	PLANDc PD SIDI maxTMAX Intercept	–0.0192 0.1858 –3.6834 –0.1013 2.9166	0.0204 0.0159 0.0327 0.0980 0.2731	0.104	0.069	0.0229
ln(nMMmax)	PLANDs PLANDc meanPPT cvPPT Intercept	0.0335 0.0175 0.1890 0.0041 –3.8780	0.0063 0.0670 0.0409 0.0916 5e-06	0.252	0.224	2e-06
ln(rLBMMmax)	PLANDs PD Intecept	–0.0895 0.2588 –1.0424	4e-06 0.0790 0.0265	0.281	0.260	2e-05
dtSAmax	PLANDwh meanPPT Intercept	0.2724 9.0870 7.0040	0.0916 4e-06 0.2662	0.318	0.297	3e-06
dtRespLB	SIDI Intercept	51.27 –17.25	0.0616 0.3397	0.202	0.152	0.0616
dtRespMM	Intercept	22.409	2e-08	–	–	–
ln(vSA)	PLANDs PD meanPPT Intercept	–0.0865 1.0201 0.4651 –4.9927	0.0127 8e-06 0.0640 2e-06	0.306	0.274	2e-05
ln(vLB)	PLANDwh PLANDc meanPPT Intercept	–0.0159 –0.0188 –0.3876 –4.3914	0.1301 0.0434 0.0007 1e-12	0.192	0.153	0.0036
ln(vMM)	PLANDc PD maxTMAX Intercept	0.0160 0.2387 0.1493 –11.4952	0.1110 0.0311 0.0703 0.0006	0.138	0.100	0.0171

None of the candidate models for dtRespLB and for dtRespMM were statistically significant.

Additionally, we identified a set of top alternative models with the AICc score higher by no more than 2 (ΔAICc < 2). This approach allowed us to identify models with smallest information loss, and thus with similar support as the optimal model. We used a conservative threshold of 2, although plausible alternative models could be identified among the models with ΔAICc ≥ 2 (43), particularly for systems for which prior knowledge is available.

Significance threshold α = 0.05 was used for all statistical analysis.

## Results

**Figure 5:**
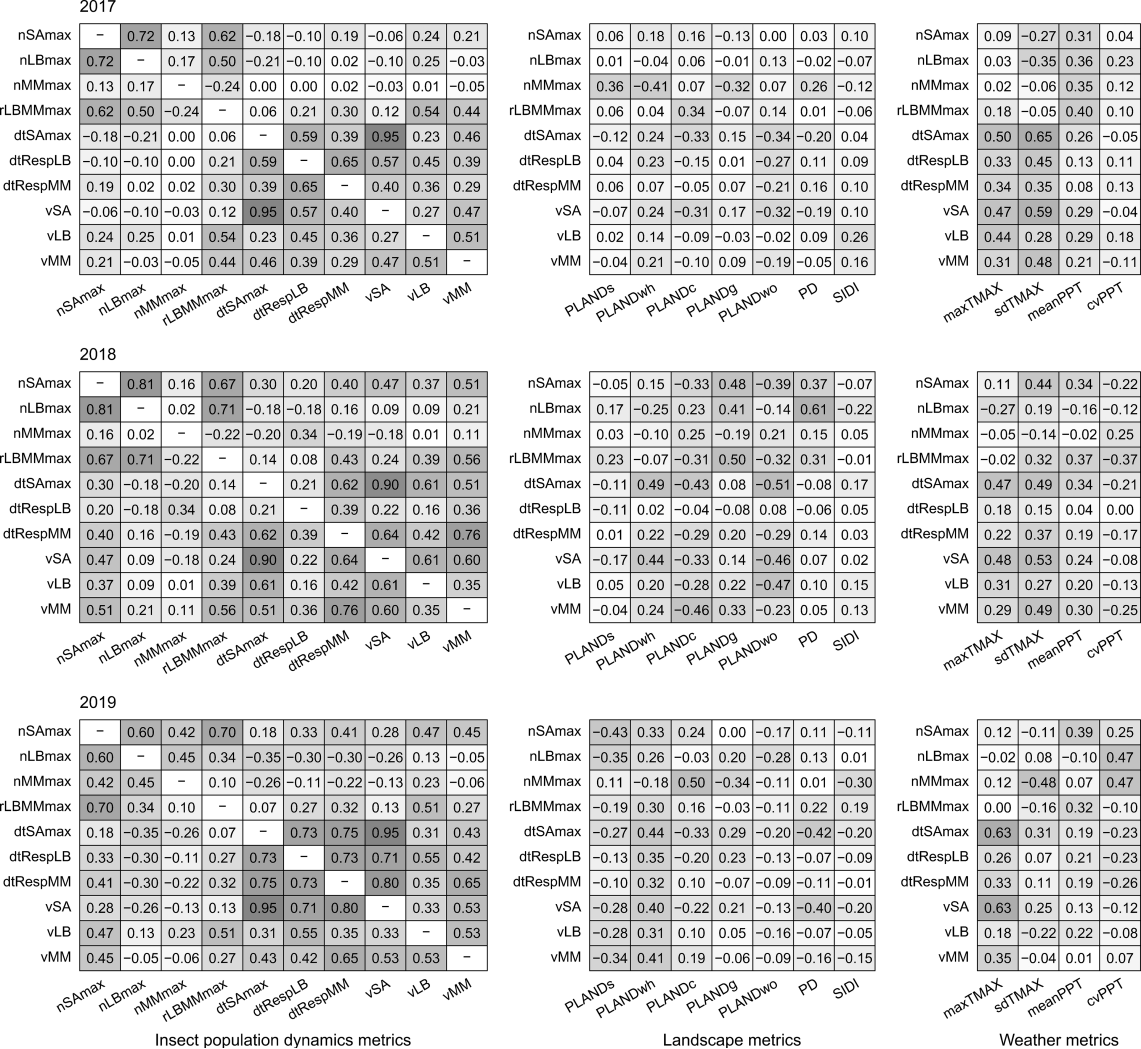
Pairwise Spearman correlation for pairs of independent variables (landscape metrics and weather metrics) and dependent variables (insect population dynamics metrics) for the years 2017–2019. Insect population dynamics metrics: nSAmax, maximum number of sorghum aphids per leaf; nLBmax, maximum number of lady beetles per leaf; nMMmax, maximum number of mummies per leaf; rLBMMmax, ratio of nLBmax and nMMmax; dtSAmax, time to maximum aphid count per leaf; dtRespLB, presumed lady beetle response time to sorghum aphid presence; dtRespMM, presumed parasitoid response time to sorghum aphid presence; vSA, speed of increase in sorghum aphid abundance; vLB, speed of increase in lady beetle abundance; vMM, speed of increase in parasitoid abundance. Landscape metrics; PLANDs, percentage of landscape (sorghum); PLANDwh, percentage of landscape (wheat); PLANDc, percentage of landscape (cotton); PLANDg, percentage of landscape (grassland, pasture, and herbaceous); PLANDwo, percentage of landscape (woodland); PD, patch density (sorghum); SIDI, Simpson’s diversity index. Weather metrics: maxTMAX, maximum value of maximum temperature; sdTMAX, standard deviation of maximum temperature; meanPPT, mean precipitation; cvPPT, coefficient of variation of precipitation. Cell shading corresponds to the level of correlation.

Correlation for pairs of dependent variables was low (Spearman correlation; r_S_ < 0.6) and varied annually, with a few exceptions (**Figure 5**). In particular, correlation through the years was consistently high between nSAmax and nLBmax (but not between nSAmax and nMMmax). Correlation was also consistently high for nSAmax and rLBMMmax, and for dtSAmax and vSA. In general, correlation was low for pairs of dependent and independent variables, with a few year-specific exceptions (e.g., for dtSAmax and sdTMAX, r_S_ = 0.65 in 2017 and r_S_ < 0.5 in 2018 and 2019).

**Figure 6:**
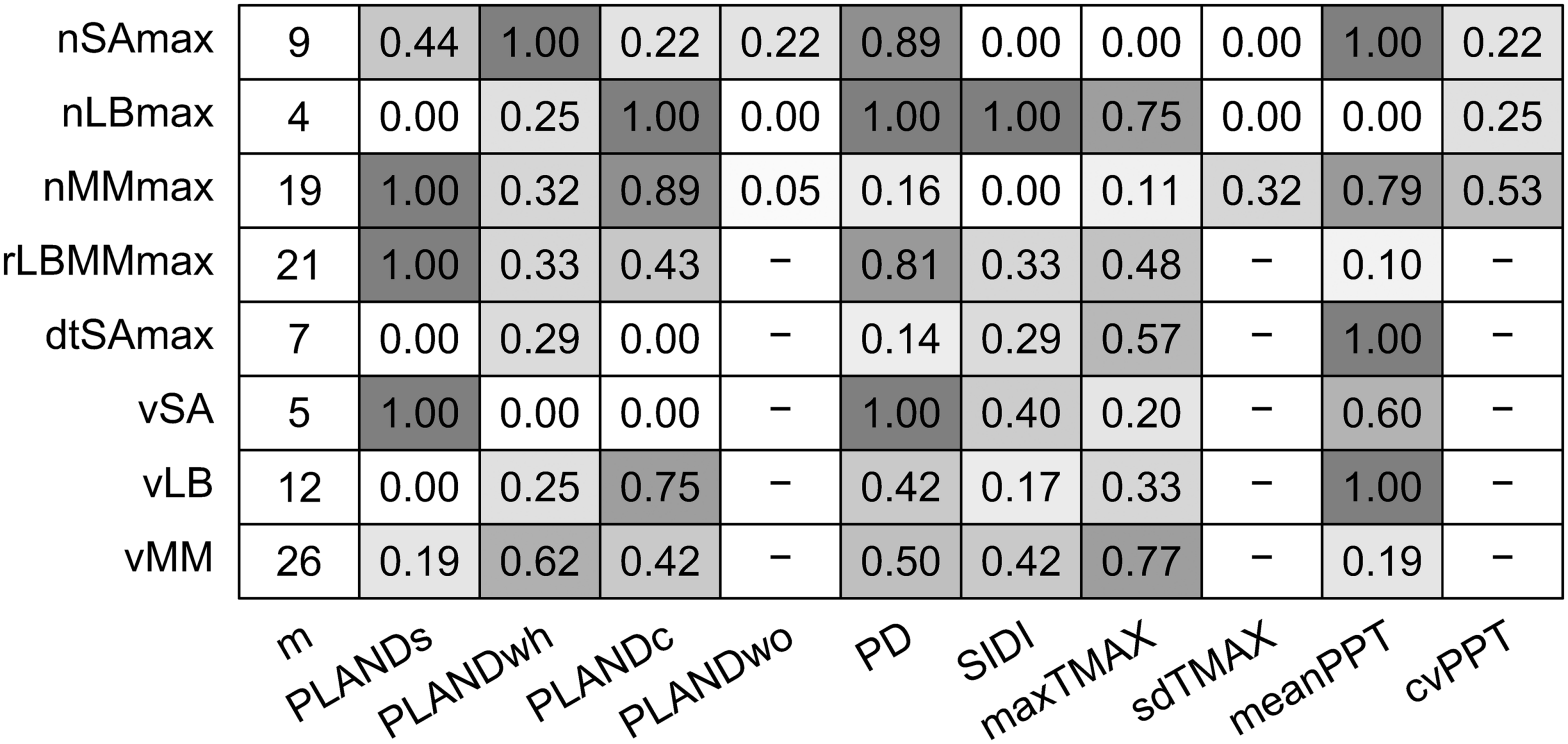
Number of alternative models with ΔAICc < 2 (m) for each of eight dependent variables (insect population dynamics metrics) and the frequency with which a given independent variable (landscape metric or weather metric) was included in the set of alternative models with ΔAICc < 2. Insect population dynamics metrics: nSAmax, maximum number of sorghum aphids per leaf; nLBmax, maximum number of lady beetles per leaf; nMMmax, maximum number of mummies per leaf; rLBMMmax, ratio of nLBmax and nMMmax; dtSAmax, time to maximum aphid count per leaf; vSA, speed of increase in sorghum aphid abundance; vLB, speed of increase in lady beetle abundance; and vMM, speed of increase in parasitoid abundance. Landscape metrics: PLANDs, percentage of landscape (sorghum); PLANDwh, percentage of landscape (wheat); PLANDc, percentage of landscape (cotton); PLANDwo, percentage of landscape (woodland); PD, patch density (sorghum); and SIDI, Simpson’s diversity index. Weather metrics: maxTMAX, maximum value of maximum temperature; sdTMAX, standard deviation of maximum temperature; meanPPT, mean precipitation; cvPPT, coefficient of variation of precipitation. Cell shading corresponds to the frequency of variable inclusion.

Considering the sets of alternative models with ΔAICc < 2, we computed the frequency with which a given independent variable was included in the set of top alternative models for each dependent variable (**Figure 6**). PLANDs was present in all the alternative models for nMMmax, rLBMMmax, and vSA. The frequency of PLANDs in the case of nSAmax was moderate, but PD was included in eight out of nine nSAmax models. PD was a commonly included variable for nLBmax, rLBMMmax, and vSA. The models for nLBmax and nMMmax had opposite tendencies regarding the inclusion of sorghum related variables PLANDs and PD. Inclusion of PLANDwh and PLANDc seemed also complimentary. PLANDwh was included in all top alternative models for nSAmax, whereas PLANDc was frequently included in models for nLBmax, nMMmax, and vLB. Except for nLBmax, the frequency of SIDI, an overall index of landscape diversity within buffers, was rather low, perhaps due to its low spatiotemporal variation (**Figure 3**). Among the weather variables, meanPPT was more commonly included than maxTMAX, especially in models for nSAmax, nMMmax, dtSAmax, and vLB. Interestingly, the frequency of meanPPT was high for nMMmax and vLB, but not for nLBmax and vMM, whereas the opposite was true for maxTMAX. The frequencies of sdTMAX and cvPPT were low to moderate. The frequencies of inclusion of independent variables align well with the sets of independent variables selected for optimal models (**Table 5**). In summary, a PLAND of a specific crop was preferred as an independent variable, and the overall diversity of the landscape (SIDI) was typically less preferred. The landscape configuration metric PD was frequently included in alternative models for most dependent variables. Moreover, the main weather effects, and not their respective variability metrics, were the preferred independent variables among the weather metrics, although their effects seemed to be either redundant or mutually exclusive rather than synergistic.

Multiple factors, and likely their interactions, have impact on the phenomena described by the ten insect metrics. In particular, in the case of metrics that describe activity of parasitoids, the number of top alternative models (ΔAICc < 2), and thus alternative combinations of landscape and weather metrics that fit data best, was relatively high – 19 for nMMmax, 21 for rLBMMmax, and 26 for vMM (**Figure 6**). Specifically, R^2^ for the optimal nMMmax model equaled 0.252 (adjusted R^2^ = 0.224; **Table 5**). To the contrary, there were only four top alternative nLBmax models with low AICc scores (**Figure 6**), but far less variability in nLBmax was explained by the optimal model, with a much lower R^2^ of 0.104 and adjusted R^2^ of 0.069 (**Table 5**). In the case of the far more host-specific sorghum aphid, the R^2^ for the optimal model was 0.151, and the adjusted R^2^ was 0.126 (**Table 5**). Despite the high correlation between nSAmax and nLBmax (but not for nSAmax and nMMmax), consistent through the years (**Figure 5**), pairwise overlap in most frequently included independent variables was surprisingly low, and PD was the only common frequently included metric in both nSAmax models and nLBmax models (**Figure 6**). For the pair nSAmax and nMMmax, meanPPT was a common frequently included metric, and for nLBmax and nMMmax the common metric was PLANDc (**Figure 6**). Pairwise correlation was also consistently high for the pairs nSAmax and rLBMMmax, and for dtSAmax and vSA. Despite the high correlation, in both cases, the frequencies of inclusion of independent variables were largely different. Judging by the frequency of independent variable inclusion, the speed of increase in sorghum aphid abundance vSA, unlike vLB and vMM, was dependent on both presence of sorghum and configuration of sorghum fields (PLANDs and PD, respectively), and to a lesser degree on meanPPT and SIDI. The speed of increase in lady beetle abundance vLB was more dependent on the presence of cotton (PLANDc) and meanPPT, although both PLANDc and PLANDwh were included in the optimal model. The speed of increase in parasitoid abundance could be explained by 26 top alternative models (ΔAICc < 2), and the frequencies of independent variable inclusion were rather moderate and more evenly distributed. PLANDwh was more commonly included variable than PLANDc in the top alternative models, although the optimal model included PLANDc. For both vLB and vMM, the more common sorghum-related variable was the landscape configuration metric PD rather than the landscape composition metric PLANDs. In summary, for a given insect category, the maximum numbers of insects and their speed of increase in abundance tend to be explained by very different sets of independent variables. In other words, different environmental factors shape different aspects of insect population dynamics in the field.

**Figure 7:**
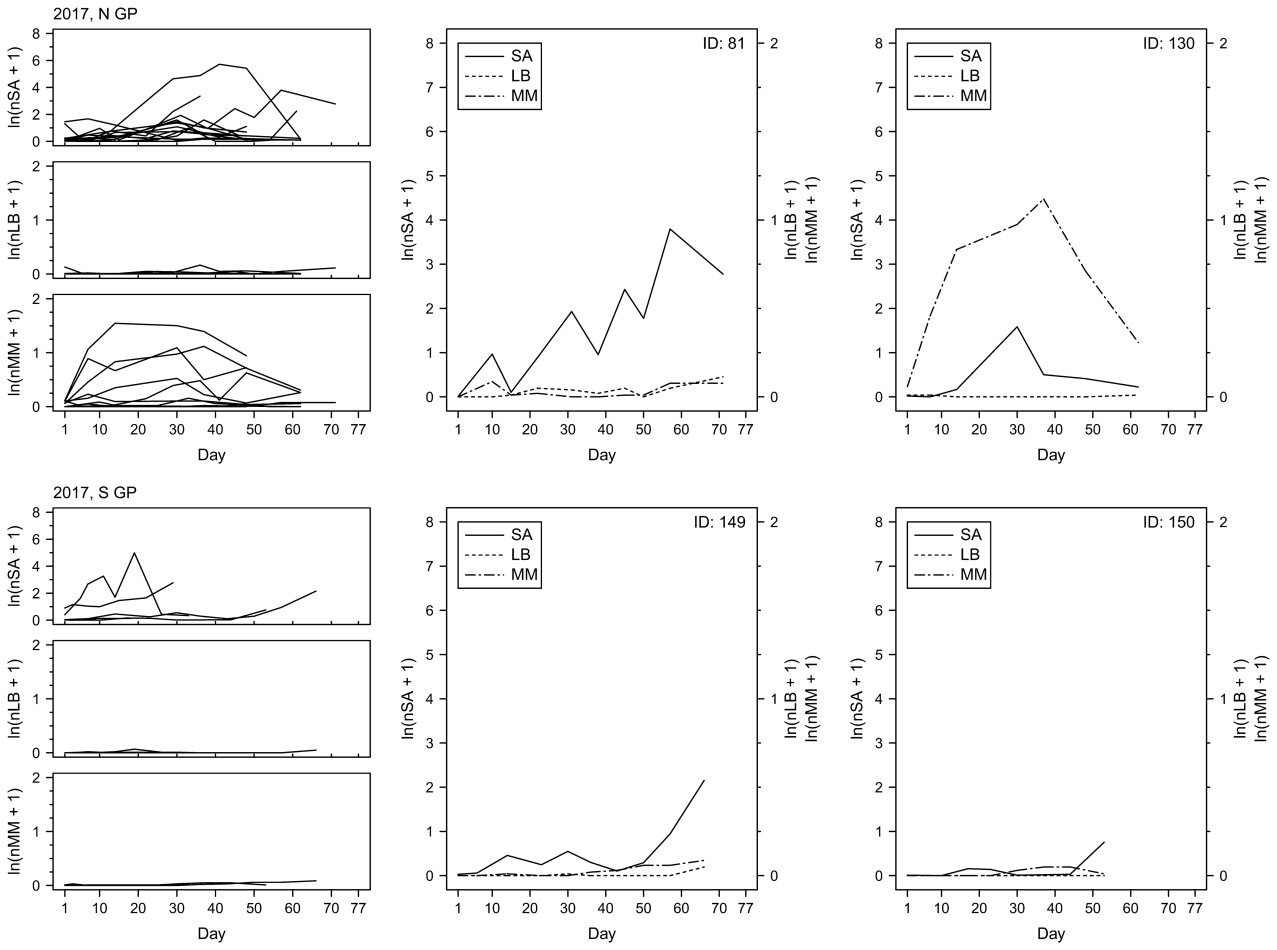
Per-leaf counts of sorghum aphids (nSA; unit: #·leaf^–1^), lady beetles (nLB; unit: #·leaf^–1^), and mummies (nMM; unit: #·leaf^–1^) observed on a given day from the day when sorghum aphids were first observed after previously not being observed until the last day when the data were collected at a given field (left column) during the year 2017. Examples of sorghum aphid and natural enemy dynamics on selected fields with the longest time of data collection (middle and right columns), with the solid, dotted, and dot-dashed lines corresponding to sorghum aphids (SA), lady beetles (LB), and mummies (MM), respectively.

**Figure 8:**
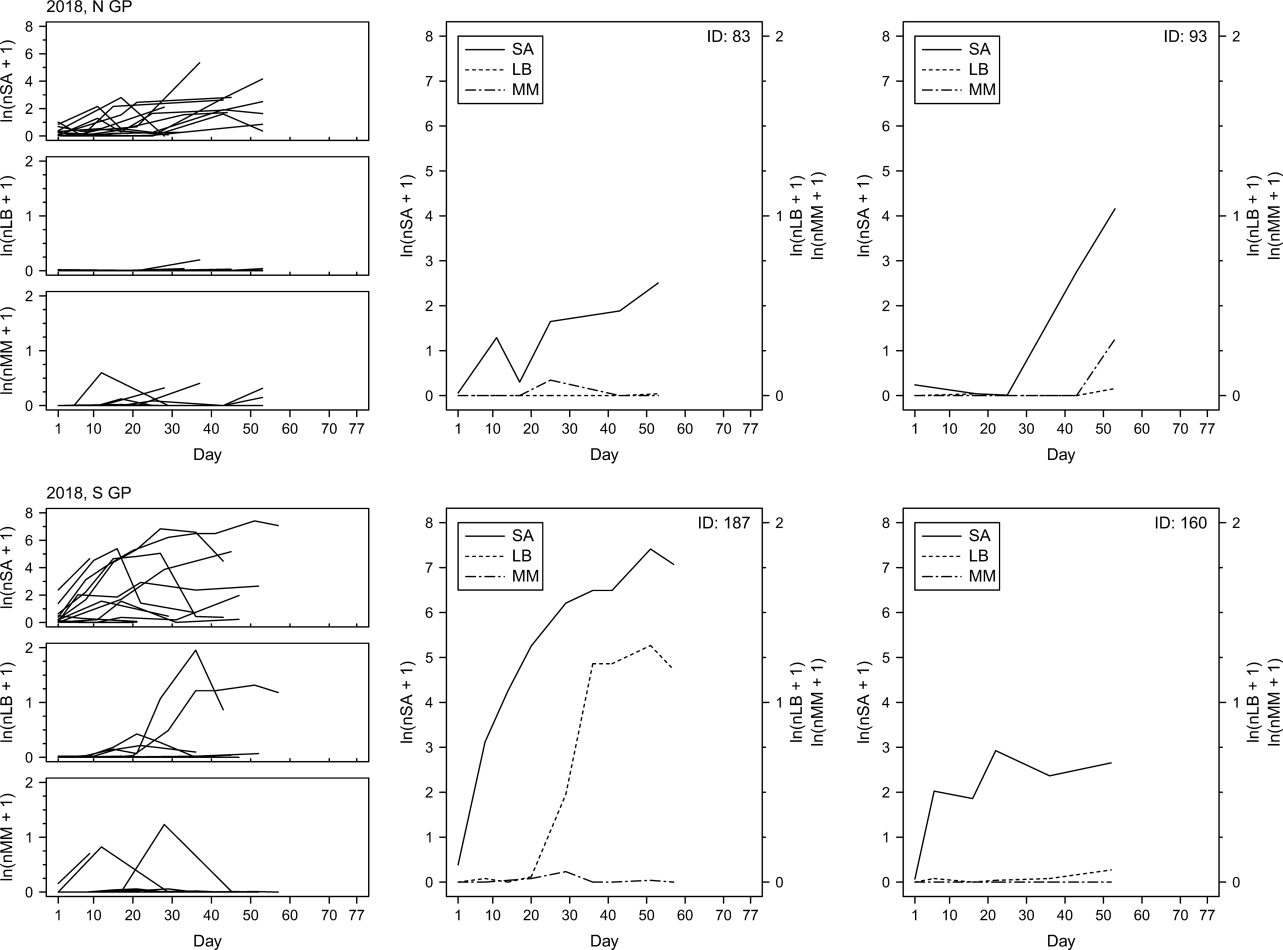
Per-leaf counts of sorghum aphids (nSA; unit: #·leaf^–1^), lady beetles (nLB; unit: #·leaf^–1^), and mummies (nMM; unit: #·leaf^–1^) observed on a given day from the day when sorghum aphids were first observed after previously not being observed until the last day when the data were collected at a given field (left column) during the year 2018. Examples of sorghum aphid and natural enemy dynamics on selected fields with the longest time of data collection (middle and right columns), with the solid, dotted, and dot-dashed lines corresponding to sorghum aphids (SA), lady beetles (LB), and mummies (MM), respectively.

**Figure 9:**
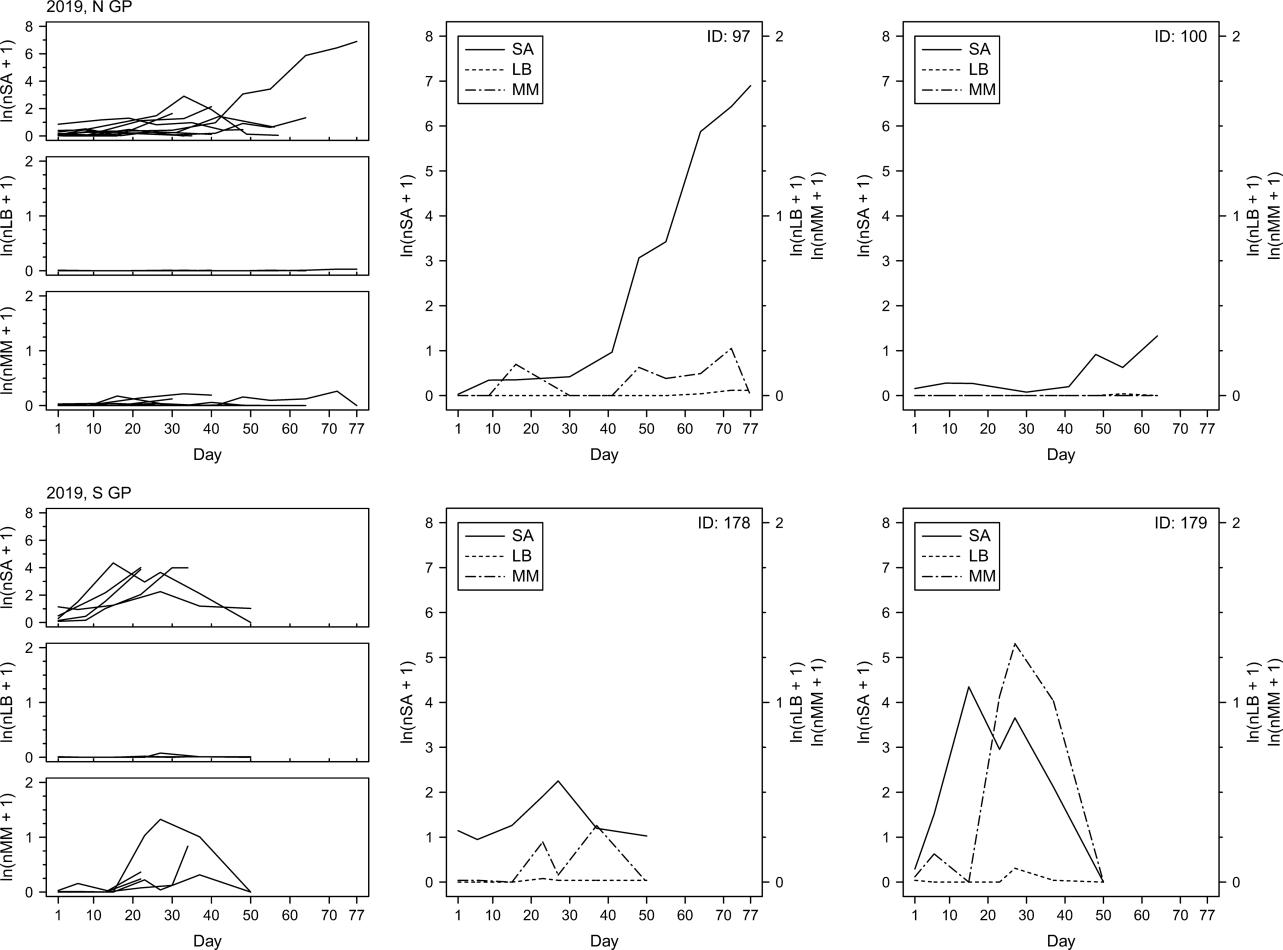
Per-leaf counts of sorghum aphids (nSA; unit: #·leaf^–1^), lady beetles (nLB; unit: #·leaf^–1^), and mummies (nMM; unit: #·leaf^–1^) observed on a given day from the day when sorghum aphids were first observed after previously not being observed until the last day when the data were collected at a given field (left column) during the year 2019. Examples of sorghum aphid and natural enemy dynamics on selected fields with the longest time of data collection (middle and right columns), with the solid, dotted, and dot-dashed lines corresponding to sorghum aphids (SA), lady beetles (LB), and mummies (MM), respectively.

To examine changes in insect counts through time, the numbers of sorghum aphids, lady beetles and mummies were plotted as a function of time (**Figures 7**–**9**). The temporal field sampling resolution allowed us to capture the emergence of patterns in insect population dynamics. The patterns in three individual examples from 2017 (**Figure 7**; IDs 81, 149 and 150) and all individual examples from 2018 and 2019 (**Figures 8**, **9**) resemble the classic predator-prey population dynamics, with spikes in numbers of predatory lady beetles and the parasitoid wasps following in time the spikes in numbers of sorghum aphids. Evident departure from this pattern can be observed in one of the examples in 2017 (**Figure 7**; ID 130), where the rapid increase of the counts of mummies seems to precede population growth of aphids. Population declines of prey and delayed declines in population count of predators and parasitoids can also be observed in some cases (e.g., year 2017, ID130; **Figure 7**), although the fate of the insects is unknown. The data does not prove causative relationships, but the patterns are consistent through the years. In general, in N GP the response of parasitoids was stronger than lady beetles during all three years, but in S GP the responses varied annually. In 2018 the response of the predators appeared stronger overall, whereas in 2019 the response of parasitoids was stronger. The responses were relatively weaker in 2017. Since each individual example reflects a series of measurements from one field, the measurements, and the resulting fluctuations, may be influenced by the stochastic nature of data sampling.

## Discussion

In this work we investigated important aspects of population dynamics of sorghum aphid and its natural enemies in the context of variable landscape and weather conditions. Our work highlights the value of longitudinal data that represent both the breadth of environmental conditions that typically change through space and their temporal variability within and between seasons. In the case of wind-borne insects, the spatial aspects of data collection should be perceived through the lens of variability in the local environmental conditions rather than the physical distances of sampling sites. Physical distance may not directly correlate with the effects of environmental conditions upon the studied system and may not be the main factor that limits distribution (44). The choice of sampling locations should account for these aspects.

Spatiotemporal effects are particularly important in the case of AWPM, in which timely and synchronized activities across distant locations are a key consideration. Spatial effects are often considered from the perspective of temporal variability (annual or seasonal variability of a given factor at a given location), but temporal effects are considered arguably less often in the context of space, both physical (differences in temporal variability of a given factor at different physical locations) and environmental (differences in temporal variability of a given factor under different environmental conditions). Clinal changes in environmental characteristics can be expected across extensive landscapes such as the Great Plains of North America, but our study shows that various measures of species responses to changing environment may show different patterns. For example, our data show that in the case of sorghum aphid the time to maximum aphid count per leaf, dtSAmax, is longer in N GP than in S and S GP, but differences in nSAmax and vSA between S, S GP and N GP are not consistent between seasons. It appears that changing local effects (and likely their interactions) have varying impact on the three sorghum aphid metrics (**Figure 2**). Similarly, maximum numbers of lady beetles and parasitoids (nLBmax and nMMmax, respectively) and their presumed response times (dtRespLB and dtRespMM, respectively) also did not show consistent spatiotemporal trends (**Figure 2**). Patterns that emerge from changes in observed insect numbers through time support these observations (**Figures 7**-**9**). As the environmental conditions change in space and time, observing one of the population metrics in isolation may lead to biased conclusions regarding species dynamics.

Considering landscape features, crop-related metrics and SIDI were the most frequently selected metrics in our regression analysis. The configuration metric PD was preferred over the related composition metric PLANDs in models for some dependent variables (e.g., for nLBmax), PLANDs was preferred over PD for some others (e.g., for nMMmax), and in some cases both were frequently used (e.g., vSA). Therefore, both descriptors of landscape should be considered in future studies. Weather effects were represented in top alternative models predominantly by maximum values of maximum temperature (maxTMAX) and by mean precipitation (meanPPT) (**Figure 6**), and the related variability metrics were selected less frequently. Analysis of longer-term data with alternative spatial resolutions may help better understand the impact of weather variability metrics, especially for those dependent variables for which these metrics were not considered (**Table 4**). Nevertheless, the importance of weather variability metrics may depend on the metric itself and on specific study design.

Our analysis highlights the complexity of ecological interactions in the field and indicates the need for a broad consideration of landscape and weather features in AWPM studies. Alternative inclusion of landscape composition and configuration metrics for various species-specific regression models indicates complementary nature of the two, but also indicates that these metrics should not be associated with a species or with a specific aspect of insect activity. Instead, the category of landscape metrics that best explains a given phenomenon likely depends on a broader environmental context. For example, although cotton and wheat habitats were more relevant for our analysis than grassland, pasture, and herbaceous and woodland habitats, PLANDwh was included in all top alternative models for maximum number of sorghum aphids nSAmax and only rarely for nLBmax and nMMmax, and PLANDc showed the opposite trends. Analysis focused on sorghum aphid alone would likely miss the effects of PLANDc on the predator-parasitoid-prey system. Moreover, the two groups of natural enemies responded differently to landscape composition and configuration features as well as to the weather conditions. Therefore, there seems to be no single metric or a set of metrics that can holistically describe the activity of sorghum aphid and of its specific natural enemy in the field. Instead, various aspects of the insect population dynamics need to be explained in light of different environmental factors. Moreover, it may be important to retain the hierarchy between the metrics as landscape components (landscape composition) may need to be considered first before landscape features (landscape configuration) can be analyzed or their effects interpreted.

The complexity of intra- and inter-specific insect dynamics in the field is also evident in the large portion of variation not explained by regression models in our study. Impact of landscape and climate and weather factors not considered here should be investigated further in future studies. For example, landscape configuration metrics for land cover categories other than sorghum may provide more insight into the effects of those categories. Deeper understanding of causative relationships between the investigated metrics is also essential, and it can only be done with thorough investigation of the studied system, and complexity of such exploration in practice may be prohibitive. Moreover, complex ecological relationships among species in the local agroecosystem may result in intricate and implicit effects. All these phenomena may extend beyond the spatial buffer in focus, a boundary that facilitates analysis but narrows analytical perspective.

Our study complements previous analytical efforts to describe sorghum aphid and its natural enemies. In a study that spanned over a similar spatial range, Brewer et al. (11) noted that the correlations between insect interactions (sorghum aphid and natural enemies) and environmental characteristics (landscape features and weather conditions) varied among the S, S GP and N GP regions. In the S region, the relationships between insects were strong but apparently independent from the environmental factors. Influences of environmental factors became apparent in S GP, where parasitoids negatively correlated with percentage of buffer occupied by cropland and lady beetle adults negatively correlated with percentages of buffer occupied by shrubland and by sorghum. In the N GP the significant correlations between insects were limited to predators but activity of parasitoids could be linked with percentages of buffer occupied by sorghum and by shrubland. Elliott et al. (26) found that percentage of buffer occupied by wooded land was significantly correlated with parasitism by *L. testaceipes* on aphids in winter wheat in Oklahoma, and that both multiple landscape composition and multiple landscape configuration metrics were significantly correlated with parasitism by *L. testaceipes*. They also found that parasitisism increased with increasing landscape diversity, indicating that *L. testaceipes* utilizes multiple habitats throughout the year. To the contrary, Elkins et al. (10) found that parasitism decreased with increase of landscape complexity. Compared to the southerly Texas Gulf Coast, the more challenging winter conditions in Oklahoma make availability of suitable winter habitats more challenging, a likely reason for the differences. Differences between these studies and our study likely stem from differences in timing and sampling locations, but also from differences in spatiotemporal scale and resolution.

**Figure 10:**
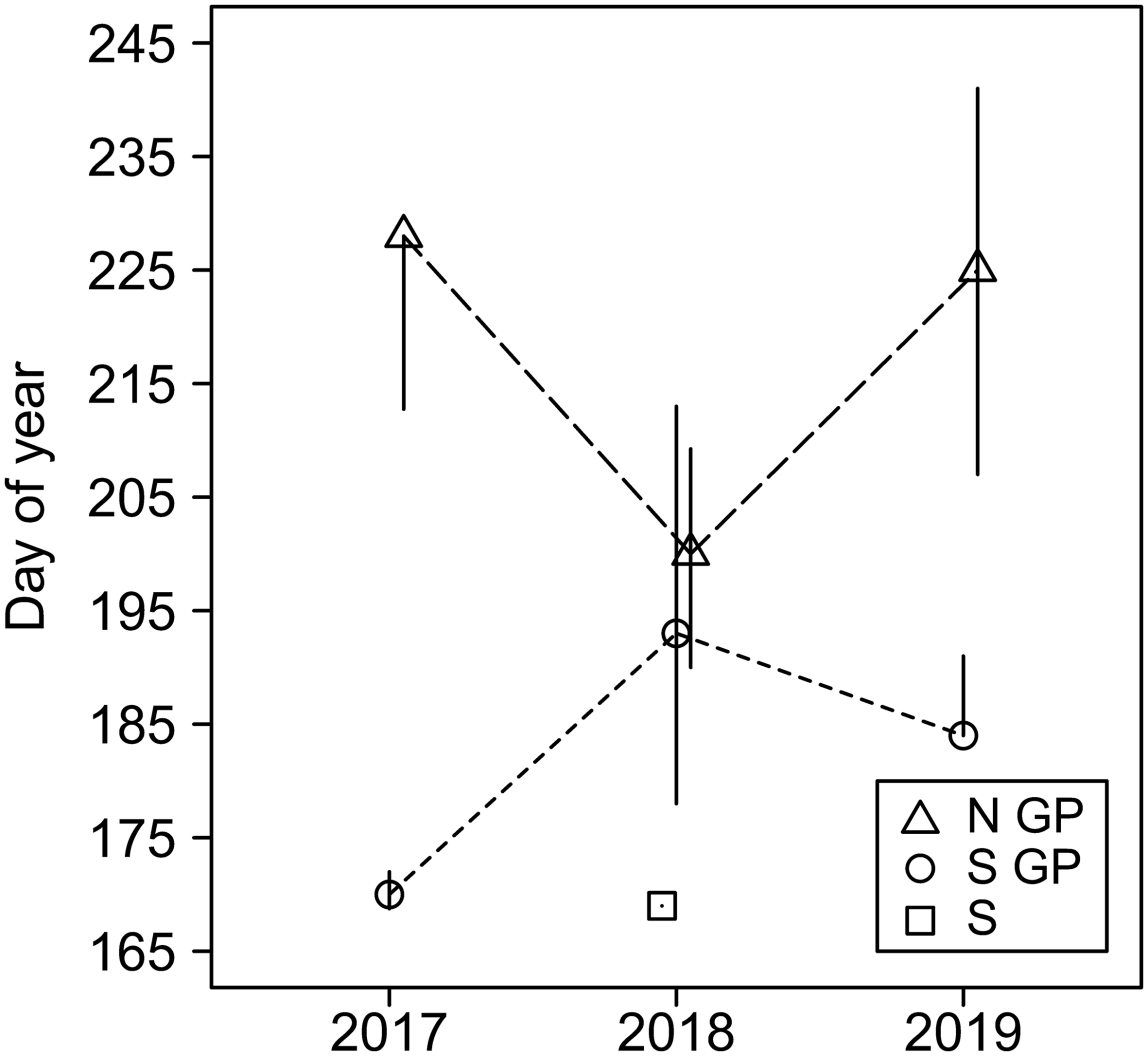
Day of first observation of sorghum aphid on sites in North Great Plain (N GP), South Great Plains (S GP), and South (S) during the years 2017–2019. Markers represent the median day of year when sorghum aphid was first observed after previously not being observed, and vertical lines represent related interquartile ranges.

Timing of field activities, such as data collection, is of high importance. Simulation modeling is one approach that could help mitigate such challenges by aiding management decisions, potentially in near-real-time (20, 21). The regional aphid infestation may be perceived as a northward movement of the “aphid invasion front,” which is limited primarily by sorghum planting dates (45). However, winters are mild in the south and outbreaks may occur locally from remnant aphid populations overwintering on rudimentary plants. Nevertheless, dates of first infestation within season may be surprisingly close across large distances, they can be highly variable within a region, and they may vary significantly annually (**Figure 10**). Data collections that start too early may result in futile efforts. Late data collections may fail to capture the first infestations. To describe some of the processes, we relied on data series with initial observations of no insects (e.g., vSA), and only those data series could be used for such estimates. Proper timing is essential to in-depth data analysis, but it may also be the most challenging factor in scheduling field activities. Computational modeling can aid field research (14) but ultimately decisions remain with practitioners.

A blend of laboratory studies, common field studies, and field data collection is needed to increase our understanding of causative effects of environmental factors on the dynamics of pest and natural enemy populations. Further investigations of pest and natural enemy interactions and impact on their respective populations need to account for temporal scale (29). A thorough understanding of the dynamics of pests and their natural enemies is critical for AWPM programs and thus for sustainable agriculture. Broader understanding of these dynamics requires holistic approaches to data collection, insect metric selection, and inclusive analysis of environmental factors in future studies. The practical application of the research results to AWPM include adjusting the timing of monitoring for pests geographically based on knowledge of historical infestation patterns, assessing the likelihood of economic infestations based on spatiotemporal features of landscapes and broader scale regions where knowledge of infestation history is applicable to predicting future infestations and their severity. In addition, finer scale attributes of landscape composition and configuration can be used to predict severity of infestations at the scale of individual fields.

## Data Availability

The raw data supporting the conclusions of this article will be made available by the authors, without undue reservation.
