# Supplementary material for: Activity of sorghum aphid and its natural enemies in the context of agroecological and weather conditions
Source: Front Insect Sci. 2025 Feb 10;5:1503044. doi: 10.3389/finsc.2025.1503044 (PMC11865913; doi:10.3389/finsc.2025.1503044)
Supplement: Supplementary file 1 [file DataSheet1.pdf]

SUPPLEMENTARY MATERIALS

SUPPLEMENTARY TABLE S1 | Overview of sampling dates and locations. Multiple fields were sampled at some sites. Nearly all fields were sampled multiple times.

State(s)	Year(s)	Range of dates	Counties	Sites	Fields	Fields resampled
TX	2015	May 21 – Oct 17	2	2	5	5
TX	2016	May 19 – Sep 11	4	4	6	6
KS, OK, TX	2017	May 8 – Oct 16	23	64	69	62
KS, OK, TX	2018	May 17 – Oct 3	20	55	60	52
OK, TX	2019	May 23 – Oct 9	15	44	47	40
KS, OK, TX	2015–2019	–	30	158	187	165

SUPPLEMENTARY TABLE S2 | Number of data points for each insect population dynamics metric in each year (values greater than 0).

Metric	2015	2016	2017	2018	2019
nSAmax	5	6	59	53	46
nLBmax	5	6	49	35	29
nMMmax	5	6	53	41	38
rLBMMmax	5	6	43	26	27
dtSAmax	0	0	24	28	18
dtRespLB	0	0	10	8	7
dtRespMM	0	0	8	14	8
vSA	0	0	24	28	18
vLB	4	0	29	20	18
vMM	4	4	35	19	18

SUPPLEMENTARY TABLE S3 | Reclassification of Cropland Data Layer landscape classes (column "Class") into 17 composite categories (column "Category"). Only classes present in buffers surrounding sites (5 km radius) sampled during the years 2015–2019 are shown. The category "Water (not waterways) and impervious surfaces" was considered background and was not used for computation of landscape metrics (termed "external background" in the software Fragstats).

**Category**	**Class**
Asteraceae	Sunflower
Brassicaceae	Canola; Turnips
Corn	Corn; Dbl Crop Barley/Corn; Dbl Crop Oats/Corn; Dbl Crop Triticale/Corn; Dbl Crop WinWht/Corn; Pop or Orn Corn
Cotton	Cotton; Dbl Crop Soybeans/Cotton; Dbl Crop WinWht/Cotton
Fallow	Barren; Fallow/Idle Cropland
Fruit trees	Cherries; Grapes; Pecans; Walnuts
Grassland, pasture, and herbaceous	Clover/Wildflower; Grassland/Pasture; Other Hay/Non Alfalfa; Switchgrass
Other crop grasses	Barley; Sod/Grass Seed; Oats; Millet; Rice; Rye
Other herbaceous, vegetables, fruits, and field crops	Herbs; Other Crops
Other Leguminosae	Alfalfa; Dry Beans; Peas; Vetch
Solanaceae	Potatoes
Sorghum	Dbl Crop WinWht/Sorghum; Sorghum
Soybean	Dbl Crop WinWht/Soybeans; Dbl Crop Soybeans/Oats; Dbl Crop Barley/Soybeans; Soybeans
Wetland	Herbaceous Wetlands; Woody Wetlands
Wheat	Spring Wheat; Triticale; Winter Wheat
Woodland	Deciduous Forest; Evergreen Forest; Mixed Forest; Shrubland
Water (not waterways) and impervious surfaces (background)	Open Water; Developed/Open Space; Developed/Low Intensity; Developed/Med Intensity; Developed/High Intensity

SUPPLEMENTARY TABLE S4 | Spearman correlation between landscape metrics for sorghum for all unique sampling locations. SIDI and SIEI are landscape-level metrics, whereas all the remaining metrics were computed at the class level.

**Year**	**PLANDs-PD**	**PLANDs-ED**	**PLANDs-PROX_MD**	**PLANDs-CLUMPY**	**PLANDs-SIDI**	**PD-SIDI**	**SIDI-SIEI**
2017	0.67	0.89	0.85	0.71	0.40	0.41	0.96
2018	0.08	0.85	0.96	0.82	0.23	–0.02	0.97
2019	0.32	0.84	0.93	0.87	0.33	0.57	0.99

SUPPLEMENTARY TABLE S5 | Insect population dynamics, landscape, and weather metrics used in the study.

Metric	Unit	Type	Definition
nSAmax	$\#\cdot leaf^{-1}$	Insect population dynamics metric	Maximum number of sorghum aphids (both alate and apterae) per leaf
nLBmax	$\#\cdot leaf^{-1}$	Insect population dynamics metric	Maximum number of lady beetles (both juvenile and adult) per leaf
nMMmax	$\#\cdot leaf^{-1}$	Insect population dynamics metric	Maximum number of mummies (combined counts of putative *A. nigritus* and putative *L. testaceipes*) per leaf
rLBMMmax	–	Insect population dynamics metric	Ratio of nLBmax and nMMmax
dtSAmax	d	Insect population dynamics metric	Time (number of days) during which aphid count per leaf increases from zero to maximum (time to maximum aphid count per leaf)
dtRespLB	d	Insect population dynamics metric	Presumed lady beetle response time to sorghum aphid presence, calculated as the number of days from the first day when sorghum aphids were observed after previously not being observed to the first day when lady beetles were observed after previously not being observed
dtRespMM	d	Insect population dynamics metric	Presumed parasitoid response time to sorghum aphid presence, calculated as the number of days from the first day when sorghum aphids were observed after previously not being observed to the first day when putative *A. nigritus* or *L. testaceipes* mummies were observed after previously not being observed
vSA	$\#\cdot leaf^{-1}\cdot d^{-1}$	Insect population dynamics metric	Increase of the number of sorghum aphids per leaf per day, from the last day when sorghum aphids were not observed to the day when their number reached maximum (speed of increase in sorghum aphid abundance)
vLB	$\#\cdot leaf^{-1}\cdot d^{-1}$	Insect population dynamics metric	Increase of the number of lady beetles per leaf per day, from the last day when lady beetles were not observed to the day when their number reached maximum (speed of increase in lady beetle abundance)
vMM	$\#\cdot leaf^{-1}\cdot d^{-1}$	Insect population dynamics metric	Increase of the number of putative *A. nigritus* and *L. testaceipes* mummies per leaf per day, from the last day when the mummies were not observed to the day

			when their number reached maximum (speed of increase in parasitoid abundance)
PLANDs	%	Landscape composition metric	Percentage of landscape class area within the buffer, calculated as a ratio of the sum of the areas of all patches of a given class (here sorghum) and the total area of the buffer (0 < PLANDs ≤ 100)
PLANDwh	%	Landscape composition metric	Percentage of landscape class area within the buffer, calculated as a ratio of the sum of the areas of all patches of a given class (here wheat) and the total area of the buffer (0 < PLANDwh ≤ 100)
PLANDc	%	Landscape composition metric	Percentage of landscape class area within the buffer, calculated as a ratio of the sum of the areas of all patches of a given class (here cotton) and the total area of the buffer (0 < PLANDc ≤ 100)
PLANDg	%	Landscape composition metric	Percentage of landscape class area within the buffer, calculated as a ratio of the sum of the areas of all patches of a given class (here grassland, pasture, and herbaceous) and the total area of the buffer (0 < PLANDg ≤ 100)
PLANDwo	%	Landscape composition metric	Percentage of landscape class area within the buffer, calculated as a ratio of the sum of the areas of all patches of a given class (here woodland) and the total area of the buffer (0 < PLANDwo ≤ 100)
PD	$(100\ \text{ha})^{-1}$	Landscape configuration/aggregation metric	Patch density, a metric that equals the number of patches of a given class (here sorghum) in the buffer per 100 ha (PD > 0, where the maximum value is constrained by spatial resolution and indicates that every other pixel is of the focal class)
SIDI	–	Landscape composition metric	Simpson's diversity index, a measure of landscape diversity that represents the probability that any two pixels selected from the buffer at random would be of different categories (0 ≤ SIDI < 1; the value of 0 indicates that the buffer contains only one patch/class, and the value approaches 1 when the number of distinct classes increases, and the area of the buffer is more equally distributed among the classes)

maxTMAX	℃	Weather metric	Maximum value of maximum temperature (based on daily maximum temperature records)
sdTMAX	℃	Weather metric	Standard deviation of maximum temperature
meanPPT	mm	Weather metric	Mean precipitation (based on daily precipitation records)
cvPPT	–	Weather metric	Coefficient of variation of precipitation
